# Identification of risk factors for heavy clinical burden in electrical workers of highlands: a cross-sectional study

**DOI:** 10.3389/fpubh.2026.1787150

**Published:** 2026-04-08

**Authors:** Qingqing Su, Qinghua He, Yonglin Fu, Qiang Lin, Haiying Xian, Tingting Liu, Miao Li, Hao Wang, Wenjin Sun, Xuan Zhang, Ling Chen, Hui Yan, Lei Chen, Fengming Luo

**Affiliations:** 1 Department of High Altitude Medicine, Center for High Altitude Medicine, West China Hospital, Sichuan University, Chengdu, Sichuan, China; 2 High Altitude Medicine Key Laboratory of Sichuan Province, West China Hospital, Sichuan University, Chengdu, Sichuan, China; 3 Center for High Altitude Medicine, Sichuan Electric Power Hospital, Chengdu, Sichuan, China

**Keywords:** clinical burden, cross-sectional study, high altitude, occupational health, risk factor

## Abstract

**Background:**

Acute and chronic mountain sickness, resulting from maladaptation to high-altitude hypoxia, poses a great health concern. This study aimed to identify risk factors for heavy symptom-based clinical burden among high-altitude electrical workers.

**Methods:**

In this cross-sectional study, 444 electrical workers stationed in the highlands of Sichuan, China, completed a symptom questionnaire and were classified as heavy (≥3 symptoms) or light (<3) clinical burden. Univariable and multivariable logistic regression identified factors associated with heavy clinical burden. Restricted cubic spline (RCS) was used to examine the nonlinear relationship between total duration of high-altitude exposure and clinical burden.

**Results:**

Of the 444 electrical workers, 218 (49.10%) had a heavy clinical burden. The most frequent symptoms were headache (50.90%), sleep disturbance (46.40%), and fatigue (45.95%). Compared to those with light clinical burden, significant differences were detected in age, ethnicity, alcohol consumption, hypertension, and altitude of residence in the heavy group (*p* < 0.05), which were all associated with heavy clinical burden in univariable analyses, but only age (OR: 1.02; 95% CI: 1.01–1.04), alcohol consumption (OR: 2.36; 95% CI: 1.55–3.61), and hypertension (OR: 1.71; 95% CI: 1.09–2.70) remained statistic significance in multivariable analyses. RCS revealed a U-shaped relationship between total duration of high-altitude exposure and clinical burden, with increased risk at <6 months and >39 months.

**Conclusion:**

Age, alcohol consumption, and hypertension are independent risk factors for heavy clinical burden in high-altitude electrical workers. Moreover, both short (<6 months) and prolonged (>39 months) duration of high-altitude exposure may confer risk for heavy clinical burden.

## 1 Introduction

With rapid socioeconomic developments in China, an increasing number of projects are being implemented in high-altitude regions, leading to greater occupational exposure to elevations above 2,500 meters. Workers in these settings often engage in high-intensity labor under extreme environmental conditions, including prolonged exposure to hypoxia, low temperatures, strong winds, and intense ultraviolet radiation (1, 2). Among these challenges, hypoxia is the most dangerous factor for health problems, and contributes to “clinical burden,” a term reflecting the complex interplay of acute and chronic physiological and pathological adaptations to high-altitude hypoxia, and encompassing a spectrum of related symptoms. Workers who are not well acclimatized may exhibit symptoms including headache, fatigue, sleep disturbances, cognitive decline, as well as severe specific complications including high-altitude pulmonary/cerebral edema, high-altitude polycythemia, and high-altitude pulmonary hypertension (3, 4). Due to the nature of their work, electrical workers frequently alternate between high-altitude and low-altitude environments, leading to intermittent hypoxic exposure. This distinctive exposure pattern makes them ideal subjects for studying acclimatization mechanisms and related health risks. Although studies on high-altitude workers have expanded in recent years (5–8), those specifically targeting electrical workers remain limited (9).

This study aims to describe clinical burden of electrical workers in high-altitude regions and identify risk factors associated with heavy clinical burden. The findings are expected to inform occupational health management strategies and also provide some evidence-based recommendations for other populations exposed to high-altitude environments.

## 2 Methods

### 2.1 Study population

In August 2024, we conducted a cross-sectional study in multiple high-altitude regions of western Sichuan, China, including Honglong (4,100 m), Litang (4,000 m), Seda (3,890 m), Xiaba (3,600 m), Ganzi (3,400 m) and Rangtang (3,285 m). Eligible participants were high-altitude electrical workers aged ≥18 years, whose altitude of residence were below 2,500 m. Exclusion criteria included severe comorbidities, such as cardio-cerebrovascular diseases, chronic respiratory diseases, etc., and a history of severe high-altitude illnesses.

This study was conducted according to the Declaration of Helsinki. The Ethics Committee of the West China Hospital of Sichuan University reviewed and approved the study protocol (No. 2023-1164). All participants provided informed consent.

### 2.2 Data collection

A cross-sectional study was conducted among 444 high-altitude electrical workers using a structured questionnaire. The survey assessed general demographic characteristics (gender, age and ethnicity) and personal lifestyle behaviors (smoking and alcohol consumption). For analysis, participants were classified as non-users (never smoker or drinker) or users (former/current smoker or drinker). It also incorporated 10 clinical maladaptation symptoms, which encompass a range of typical adverse reactions to high-altitude exposure. The 10 clinical maladaptation symptoms included dizziness/light-headedness, fatigue/weakness, gastrointestinal upset, breathlessness and/or palpitations, sleep disturbance, cyanosis, dilatation of veins, paresthesia, headache, tinnitus. Participants with ≥3 symptoms were classified into the heavy clinical burden group, while those with <3 symptoms were classified into the light clinical burden group.

Physical examinations included measurements of blood pressure (BP), waist circumference (WC), hip circumference (HC), height, weight, heart rate (HR), and oxygen saturation (SaO_2_). Body mass index (BMI) was calculated as weight in kilograms (kg) divided by height squared (m^2^). BP was measured using sphygmomanometer (Yuyue Medical Equipment Co., Ltd., China), with an appropriately sized cuff selected according to the upper arm circumference. After a standardized rest period of at least 5 min, BP was measured twice in a seated position, with the average value calculated from two measurements. HR and SaO_2_ were recorded after 10 min of rest using a pulse oximeter (Yuyue Medical Equipment Co., Ltd., China). Hypertension was defined as a systolic BP ≥ 140 mmHg and/or a diastolic BP ≥ 90 mmHg.

All the data were collected on-site at the high-altitude work locations in Sichuan Province. The participants completed the questionnaire after working at high altitudes for at least 1 week. Current symptoms were assessed at the time of clinical evaluation through standardized interview and physical examination by physicians.

### 2.3 Statistical analyses

Continuous variables were tested for normality using the Shapiro–Wilk test, which indicated non-normal distributions for all variables. So, non-normally distributed data were described as median (interquartile range), while categorical variables were presented as frequency and percentage (*n*, %). Baseline characteristics between the heavy and light clinical burden groups were compared using the Mann–Whitney U test for continuous variables and the Chi-square test for categorical variables. Univariable and multivariable binary logistic regression analyses were performed to identify the risk factors associated with the heavy clinical burden, with results expressed as adjusted odds ratios (OR) and 95% confidence intervals (CI). Three multivariable regression models were constructed with stepwise adjustment for covariates. Model 1 was a crude model with no covariates adjusted. Model 2 was adjusted for basic demographic variables. Model 3 was further adjusted for a comprehensive set of covariates. The final multivariable model included 11 predictor variables, and the heavy clinical burden group provided 218 events, yielding approximately 20 events per predictor, suggesting that our model estimates are likely to be stable. Restricted cubic spline (RCS) was performed to evaluate the nonlinear association between total duration of high-altitude exposure (≤60 months) and clinical outcomes, with results visualized as smoothed curves with 95% confidence bands.

## 3 Results

### 3.1 Clinical characteristics in the heavy and light clinical burden groups

The final analysis included 444 electrical workers, with 218 (49.10%) classified into the heavy clinical burden group (≥3 symptoms) and 226 (50.90%) into the light clinical burden group (<3 symptoms).

The clinical characteristics of the heavy and light clinical burden groups were described in the Table 1, including age, gender, ethnicity, BMI, SaO_2_, HR, smoking, alcohol consumption, hypertension, total duration of high-altitude exposure, altitude of residence and altitude of workplace. However, statistically significant differences between the two groups were observed in age, ethnicity, alcohol consumption, hypertension and altitude of residence (*p* < 0.05).

**Table 1:** Baseline characteristics of electrical workers and risk factors associated with heavy clinical burden.

Variables	Baseline characteristics				Binary logistic regression		
	Total (444)	Heavy clinical burden (218)	Light clinical burden (226)	*p*-value	Model 1 OR, (95% CI)	Model 2 OR, (95% CI)	Model 3 OR, (95% CI)
Age	37.0 (29.0, 47.0)	38.0 (31.0, 48.0)	35.0 (27.0, 45.8)	<0.001*	1.03 (1.01 ~ 1.05)	1.03 (1.01 ~ 1.04)	1.02 (1.00 ~ 1.04)
Gender				0.999			
Male	426 (95.95)	209 (95.87)	217 (96.02)		ref	ref	ref
Female	18 (4.05)	9 (4.13)	9 (3.98)		1.04 (0.40 ~ 2.71)	1.07 (0.40 ~ 2.83)	1.40 (0.51 ~ 3.92)
Ethnic Han				0.025*			
No	148 (33.33)	61 (27.98)	87 (38.50)		ref	ref	ref
Yes	296 (66.67)	157 (72.02)	139 (61.50)		1.61 (1.08 ~ 2.41)	1.38 (0.91 ~ 2.10)	1.20 (0.71 ~ 2.03)
BMI	23.3 (21.7, 25.0)	23.4 (22.0, 25.4)	23.1 (21.6, 24.8)	0.123	1.06 (0.99 ~ 1.13)	1.03 (0.95 ~ 1.10)	0.99 (0.91 ~ 1.07)
SaO_2_	91.0 (89.0, 92.0)	91.0 (89.0, 93.0)	90.5 (89.0, 92.0)	0.356	1.00 (0.94 ~ 1.06)	1.01 (0.95 ~ 1.07)	1.01 (0.95 ~ 1.07)
HR	84.0 (78.0, 90.0)	85.5 (78.2, 90.0)	84.0 (76.0, 90.0)	0.097	1.02 (1.00 ~ 1.03)	1.02 (1.00 ~ 1.04)	1.02 (1.00 ~ 1.04)
Smoking				0.556			
No	211 (47.52)	100 (45.87)	111 (49.12)		ref	ref	ref
Yes	233 (52.48)	118 (54.13)	115 (50.88)		1.14 (0.79 ~ 1.66)	1.24 (0.84 ~ 1.83)	0.89 (0.58 ~ 1.37)
Alcohol consumption				<0.001*			
No	208 (46.85)	80 (36.70)	128 (56.64)		ref	ref	ref
Yes	236 (53.15)	138 (63.30)	98 (43.36)		2.25 (1.54 ~ 3.31)	2.27 (1.54 ~ 3.36)	2.36 (1.55 ~ 3.61)
Hypertension				0.019*			
No	319 (71.85)	145 (66.51)	174 (76.99)		ref	ref	ref
Yes	125 (28.15)	73 (33.49)	52 (23.01)		1.69 (1.11 ~ 2.57)	1.64 (1.08 ~ 2.52)	1.70 (1.06 ~ 2.73)
Total duration of high-altitude residence				0.421			
≤12 months	258 (58.11)	122 (55.96)	136 (60.18)		ref	ref	ref
>12 months	186 (41.89)	96 (44.04)	90 (39.82)		1.19 (0.82 ~ 1.74)	1.10 (0.75 ~ 1.61)	1.05 (0.69 ~ 1.59)
Altitude of residence				0.048*			
≤1,500 m	301 (67.79)	158 (72.48)	143 (63.27)		ref	ref	ref
>1,500 m	143 (32.21)	60 (27.52)	83 (36.73)		0.65 (0.44 ~ 0.98)	0.83 (0.50 ~ 1.37)	0.76 (0.44 ~ 1.31)
Altitude of workplace				0.919			
≤4,000 m	338 (76.13)	165 (75.69)	173 (76.55)		ref	ref	ref
>4,000 m	106 (23.87)	53 (24.31)	53 (23.45)		1.05 (0.68 ~ 1.62)	1.10 (0.71 ~ 1.72)	1.02 (0.61 ~ 1.71)

Data are presented as median (interquartile range) for continuous variables and number (percentage) for categorical variables.

Model 1: No covariates were adjusted.

Model 2: Age, Gender, Ethnic Han were adjusted.

Model 3: Age, Gender, Ethnic Han, BMI, SaO_2_, HR, Smoking, Alcohol consumption, Hypertension, Total duration of high-altitude residence, Altitude of residence and Altitude of workplace were adjusted.

**Figure 1:**
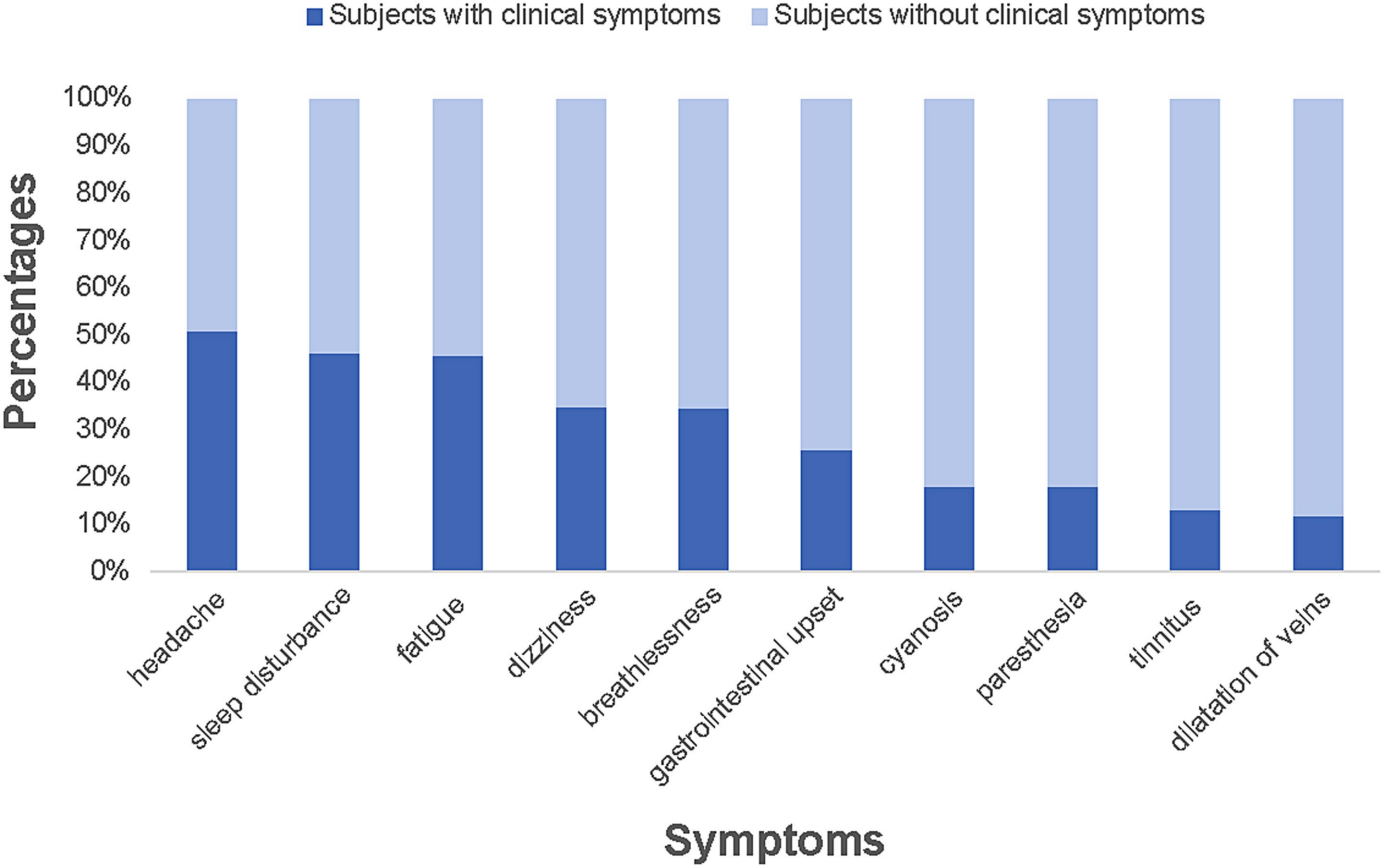
Percentages of symptoms in the high-altitude electrical workers. The bar chart illustrates the percentages of individuals with the 10 clinical symptoms in the high-altitude electrical workers. The percentages for the individuals with clinical symptoms were shown in dark blue, while those without clinical symptoms were shown in light blue.

The symptom profiles were overall depicted in Figure 1. The five most prevalent symptoms were headache (*n* = 226, 50.90%), sleep disturbances (*n* = 206, 46.40%), fatigue/weakness (*n* = 204, 45.95%), dizziness/light-headedness (*n* = 155, 34.91%), and breathlessness and/or palpitations (*n* = 154, 34.68%).

### 3.2 Risk factors associated with heavy clinical burden by univariable and multivariable binary logistic regression analyses

Univariable and multivariable binary logistic regression analyses were performed to identify factors independently associated with heavy clinical burden (Table 1). In the univariable analyses, age, ethnicity, alcohol consumption, and hypertension were positively associated with heavy clinical burden, whereas altitude of residence showed a negative association. However, in the multivariable analyses, only age (OR: 1.02; 95% CI: 1.01–1.04), alcohol consumption (OR: 2.36; 95% CI: 1.55–3.61), and hypertension (OR: 1.71; 95% CI: 1.09–2.70) remained significantly associated with heavy clinical burden (*p* < 0.05).

### 3.3 Trends of symptom prevalence over total duration of high-altitude exposure

**Figure 2:**
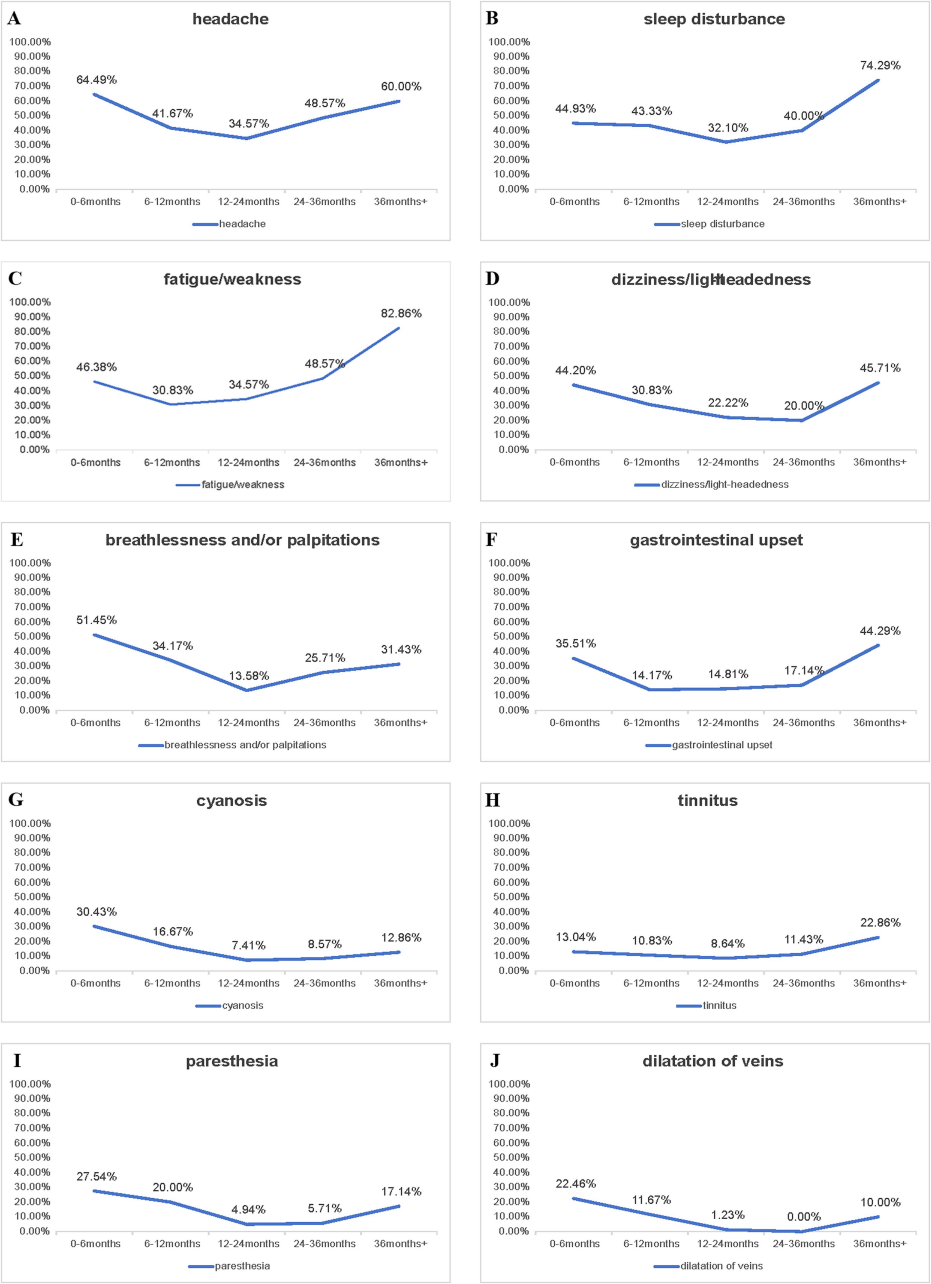
Trends of symptom prevalence over total duration of high-altitude exposure. Line graphs illustrate the trends of prevalence rates of 10 clinical symptoms **(A–J)** in the electrical workers over different total duration of high-altitude exposure (0–6, 6–12, 12–24, 24–36 and >36 months).

The prevalence (%) of 10 clinical symptoms across five groups of electrical workers stratified by total duration of high-altitude exposure (0–6, 6–12, 12–24, 24–36 and >36 months) were indicated in Figure 2. The line graphs showed an overall trend of falling first and then rising in individual prevalence rate of clinical symptom.

### 3.4 Nonlinear association between total duration of high-altitude exposure and clinical burden by RCS

**Figure 3:**
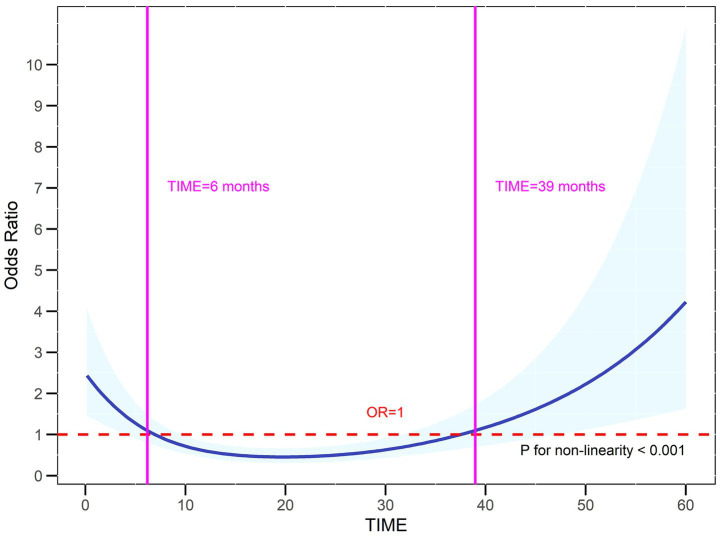
Nonlinear association between total duration of high-altitude exposure and clinical burden by RCS. RCS illustrates a non-linear association between total duration of high-altitude exposure and clinical burden. The U-shaped blue curve represents the smoothed relationship, and the light blue shadow represented its 95% confidence intervals.

As demonstrated in Figure 3, a clear U-shaped relationship was existed between total duration of high-altitude exposure and clinical burden. Workers with less than 6 months of high-altitude exposure had higher odds of high clinical burden, which may reflect acute hypoxic responses following initial exposure to high altitude. Interestingly, those with over 39 months of high-altitude exposure also showed elevated risks for heavy clinical burden, possibly reflecting maladaptation or cumulative damage from chronic hypoxia.

## 4 Discussion

High-altitude electrical workers might have some special symptoms in response to hypoxic environment. High-altitude exposure induces a series of physiological and pathological changes (10, 11). Based on these changes, we proposed the term “clinical burden” to quantify the cumulative symptomatic load in this occupational population. In this study, age, alcohol consumption, and hypertension were identified as risk factors for heavy clinical burden, and a U-shaped relationship was notably observed between total duration of high-altitude exposure and clinical burden.

The association between age and altitude-related diseases is inconsistent across the literature. In the previous studies, some have identified age as a risk factor (12–14), some have even indicated a protective effect of older age (15), while others have reported no significant relationship (16, 17). In contrast, our study assessed the heavy clinical burden—defined by the presence of multiple concurrent symptoms. The positive correlation between older age and heavy clinical burden may reflect the cumulative physiological stress brought about by aging under hypoxic conditions, including damage to cardiovascular capabilities. This explanation is supported by relevant study (18). Although the statistical significance of the age factor is limited (odds ratio: 1.02; 95% confidence interval: 1.01–1.04), older individuals should be vigilant about the heavy clinical burden.

Alcohol consumption can increase the risk of AMS (15, 19), as it may inhibit acute ventilation adaptation to mild hypoxia and impair judgment (15). Ethanol interacts with gamma-aminobutyric acid type A receptors to induce hyperpolarization of brainstem respiratory neurons, thereby suppressing central respiratory drive, blunting ventilatory responses to hypercapnia and hypoxia, and ultimately causing respiratory depression with reduced ventilatory efficiency (20, 21). These mechanisms may underlie the observed link between alcohol consumption and elevated clinical burden.

The association between hypertension and heavy clinical burden remains complex. Some studies have shown that there is no relationship between hypoxia and blood pressure (22, 23). However, hypoxia has been shown to elevate heart rate and stimulate erythropoiesis (11, 24, 25), which results in increased blood viscosity (26). These hypoxia-induced physiological changes might aggravate the condition of hypertensive patients, leading to symptoms such as headache, dizziness, gastrointestinal upset, and dyspnea. Therefore, the previously existing hypertension may have an impact on the clinical burden, but further research is needed to confirm these relationships.

Of note, ethnicity and altitude of residence were shown to be associated with heavy clinical burden in the univariable analyses, although these associations were not significant after multivariable adjustments. Among the electrical workers, the two largest ethnic groups were Han (*n* = 296) and Yi (*n* = 124) populations. Existing evidence suggested that like Tibetan, Yi individuals were part of the Tibeto-Burman lineage (27). They had greater adaptation to hypoxia than Han individuals, potentially related to long-term high-altitude exposure and genetic adaptation (28). Given these distinctions, Han individuals may face a comparatively heightened risk of elevated clinical burden in hypoxic settings. Altitude acclimatization requires sufficient time for physiological adaptation. When lower altitude residents (≤1,500 m) ascend rapidly to high altitude, they often fail to acclimatize adequately (29). This inadequate acclimatization frequently manifested as AMS, characterized by symptoms such as headache, fatigue, and sleep disturbances. Additionally, previous literature has reported that an increase in altitude gradient is associated with the incidence of altitude sickness (30), but no significant association was observed between altitude of workplace and clinical burden in this study. This may be affected by a variety of confounding factors and sample size.

Moreover, the total duration of high-altitude exposure may influence the severity of clinical symptom burden. Physiological acclimatization to hypoxia typically requires weeks to months (31), during which acute symptoms may gradually improve, and prolonged exposure to hypoxic environments may increase the risk of CMS (32). In this study, although no statistically significant association was revealed between total duration of high-altitude exposure and symptom-based clinical burden, RCS analysis demonstrated a clear U-shaped relationship. This pattern indicated three distinct physiological phases in response to high-altitude hypoxia: (i) In the initial phase (<6 months), clinical burden increased as the body experiences acute hypoxic stress before full acclimatization, which was consistent with an acclimatization period of about 6 months (33). (ii) From 6 to 39 months, clinical burden went down to its lowest level, reflecting effective compensatory adaptations that reduced symptoms. (iii) Beyond 39 months, clinical burden rose up again, potentially indicating the onset of decompensation due to sustained hypoxic exposure, which aligned with the known progression of CMS, characterized by symptoms such as headache, breathing difficulties, and sleep disorders (32). Therefore, prolonged and sustained hypoxic exposure was a primary driver underlying the development of these chronic altitude-related symptoms (34, 35). So, we propose around 39 months of total duration of high-altitude exposure as a candidate threshold for intensified clinical monitoring, which could be a practical time frame to guide health surveillance and rotational strategies in high-altitude migrants. However, these findings should be interpreted with caution and require validation in larger cohorts with more precise assessment.

Smoking was not significantly associated with heavy clinical burden in our study, which is consistent with other studies (36). However, there were also studies indicating that smoking is associated with altitude sickness (37). Given the effects of smoking on pulmonary function and cardiovascular health (38, 39), it remains a critical concern. We also recommend regular blood pressure monitoring, alcohol consumption control and closer observation of older workers with multiple risk factors. These simple strategies may help identify at-risk individuals and reduce the burden on high-altitude medical services.

Although this study provided valuable insights into high-altitude clinical burden, several limitations must be acknowledged. First, the cross-sectional design precluded causal inference. Second, the study population was limited to electrical workers, which might affect the generalizability of the findings. Third, the self-reported data might induce potential information bias. Fourth, the small female cohort limited gender-based analysis. Finally, occupational confounders, such as labor intensity and cumulative employment duration, were not comprehensively evaluated. So, future studies should include other high-altitude occupational groups, integrate biomarker analysis to validate the results, and a larger sample size is warranted to improve the predictive accuracy of the model.

## 5 Conclusion

This cross-sectional study found that age, alcohol consumption, and hypertension may be independent risk factors for heavy clinical burden in high-altitude electrical workers. In addition, total duration of high-altitude exposure (<6 months or >39 months) may also confer risk for heavy clinical burden.

## Data Availability

The raw data supporting the conclusions of this article will be made available by the authors, without undue reservation.
